# Response to Morita et al., Re: *Defining* “*Continuous Deep Sedation” Using Treatment Protocol* (DOI: 10.1089/pmr.2021.0058)

**DOI:** 10.1089/pmr.2022.0020

**Published:** 2022-06-07

**Authors:** Robert G. Twycross

**Affiliations:** Emeritus Clinical Reader in Palliative Medicine, Oxford University, Oxford, United Kingdom.

Morita et al. are to be thanked for their attempt to bring greater clarity to the subject of sedation at the end of life, more specifically in relation to continuous deep sedation (CDS) as an extreme (“far-reaching”) form of palliative sedation.^1^ Of the four types they describe, three are variations of proportional sedation, only the fourth is irreversible CDS from start to death. They comment that this “may be an essentially separate medical practice from the other three types.” I agree, and suggest that it falls outside the definition of palliative sedation.

As emphasized in The Principles of Revised Clinical Guidelines about Palliative Sedation Therapy of the Japanese Society for Palliative Medicine, palliative sedation should, like all therapeutic measures, be proportional to need.^2^ Even in a catastrophic situation, for example, a massive cancer-related arterial hemorrhage, proportionality is still relevant even though the initial dose of sedatives will almost certainly be higher than those used in less dramatic circumstances.

Morita et al. suggest that the inclusion of “intent to reduce consciousness” in earlier descriptions of palliative sedation should be abandoned because of the difficulty in evaluating this.^1^ However, there is always intention in therapeutics, and in this situation the intention is to palliate suffering, not to shorten life. As noted in the revised Japanese guidelines, “It is ethically unacceptable to enforce palliative sedation intended to shorten life, which is different from foreseeing the foreshortening of life.”^2^ Morita et al. suggest that we should speak of “treatment goal” rather than “intention.” For me, the two terms are synonymous, but the former increases clarity and thus is preferable.

For most people, a reducing level of consciousness is part of the natural dying process: progressive organ failure results in the accumulation of toxic metabolites, leading to depression of both cerebral cortex and brainstem. Sedatives will tend to accelerate and potentiate both a reduction in level of consciousness and cardiorespiratory failure. In those very close to death, the relative contributions of sedative medication and of natural dying to the reduced consciousness are impossible to determine.

In Japan, the estimated prognosis of patients requiring CDS typically seems to be only a few days.^2,3^ This suggests that the typical clinical context for CDS in Japan could be different from, for example, that in The Netherlands, even though guidelines in both countries permit CDS in patients with an estimated prognosis of less than or equal to two weeks. Thus, for comparative purposes, it is essential for any treatment protocol used for monitoring and research purposes to record the physical status of the patients when CDS is started, and the time until death.

It is also important that research is undertaken to identify the reasons for the gross disparity between the incidence of CDS in some nationwide studies (15–25%), particularly in countries where medically assisted dying is permitted (euthanasia, assisted suicide), and that reported by some palliative care services (1–3%).^4^

## Abbreviation Used

CDS: continuous deep sedation
